# PLGA-PEG-c(RGDfK)-*Kushenol* E Micelles With a Therapeutic Potential for Targeting Ovarian Cancer

**DOI:** 10.1049/nbt2/7136323

**Published:** 2024-11-29

**Authors:** Xue Chen, Haopeng Wan, Lijuan Lu, Ran Li, Bo Sun, Juan Ren

**Affiliations:** ^1^Department of Traditional Chinese Medicine, Nanxiang Branch of Ruijin Hospital, Shanghai 201802, China; ^2^Department of Gynecology, Suzhou TCM Hospital Affiliated to Nanjing University of Chinese Medicine, Suzhou 215000, China; ^3^School of Pharmacy, Jiangsu University, Zhenjiang 212001, China; ^4^Department of Gynecology, Fangta Traditional Chinese Medicine Hospital of Songjiang District of Shanghai, Shanghai 201699, China; ^5^Clinical Medical Center of Oncology, Shanghai Municipal Hospital of Traditional Chinese Medicine, Shanghai University of Traditional Chinese Medicine, Shanghai 200241, China

**Keywords:** c(RGDfK), *Kushenol E*, ovarian cancer, targeting micelle

## Abstract

**Background:** As a naturally derived inhibitor of autophagy, Kushenol E (KE) is a biprenylated flavonoid and is isolated from *Sophora flavescens*, which has been used for the treatment of cancer, hepatitis, and skin diseases. However, KE, as a poorly soluble drug, exhibited strong autophagy regulating activity in in vitro cancer cell lines, but no related studies have reported its antiovarian cancer property. Therefore, it is very beneficial to enhance the antineoplastic properties of KE by establishing an ovarian tumor-targeting nanoparticle system modified with tumor-homing c(RGDfK) peptides.

**Materials and Methods:** In the current study, poly(lactic-co-glycolic acid)-poly(ethylene glycol)-modified with cyclic RGDfK peptide (PLGA-PEG-c(RGDfK))-KE micelles (PPCKM) were prepared to overcome the poor water solubility of KE to meet the requirement of tumor-active targeting. The effect of PPCKM on ovarian cancer was evaluated on SKOV-3 cells and xenograft models in BALB/c nude mice.

**Results:** The PPCKM showed a higher drug cumulative release ratio (82.16 ± 7.69% vs. 34.96 ± 3.05%, at 1.5 h) with good morphology, particle size (93.41 ± 2.84 nm), and entrapment efficiency (89.7% ± 1.3%). The cell viability, migration, and apoptosis analysis of SKOV-3 cells demonstrated that PPCKM retained potent antitumor effects and promoted apoptosis at early and advanced stages with concentration-dependent. Based on the establishment of xenograft models in BALB/c nude mice, we discovered that PPCKM reduced tumor volume and weight, inhibited proliferating cell nuclear antigen (PCNA) and Ki67 expression, as well as promoted apoptosis by targeting the tumor site.

**Conclusion:** The findings in this study suggest that PPCKM may serve as an effective therapeutic option for ovarian cancer.

## 1. Introduction

Ovarian cancer is ranked as the third gynecological cancer in terms of frequency after cancer of the cervix and uterine but has the highest death rate [1]. The 5-year survival rate of the disease is roughly 47% [2]. A total of 313,959 new cases of the disease were recorded globally in 2020, with the age-standardized rates of incidence of ovarian cancer being 6.6 per 100,000 [3]. Characteristics of the disease include poor prognosis, late-stage presentation, and high mortality rates. The high ovarian cancer mortality rate is driven by factors such as diagnosis of the disease in the advanced stage of the disease because of lack of proper screening tests, delayed onset of symptoms, asymptomatic, and secret tumor growth [4]. Thus, the disease is referred to as a silent killer because its early detection is difficult because of vague symptoms [1]. Based on existing knowledge, no pathological process of ovarian cancer is widely accepted to explain the onset and development of the disease [5]. The underlying reason may be the heterogeneous nature of this type of cancer [6]. Response to systemic treatment and/or surgery is affected by the heterogeneous nature of the disease, which promotes acquired and inherent resistance to drugs [7]. At present, ovarian cancer is therapeutically managed through multi-disciplinary strategies such as chemotherapy, debulking surgery, and radiotherapy, albeit this treatment option being rarely used [8]. Among these, the main therapeutic option for ovarian cancer is cytoreductive surgery, whilst chemotherapies like cytotoxic taxane and platinum can be given to the patients afterward. Initially, most ovarian cancer patients respond to the aforementioned treatment options, but in the advanced stage individuals, the disease is likely to recur within an average of 16 months. Therefore, there is an urgent need to develop more efficacious treatment strategies to prevent and delay ovarian cancer.

Scientists have, over the years, developed plants and their derivatives into an effective anticancer agent. The derivatives of plants that have shown strong anticancer effects included but not limited to etoposide, irinotecan, paclitaxel, podophyllotoxin, topotecan, vinblastine, and vincristine [9]. Available literature suggests that various active ingredients of some medicinal plants may treat ovarian cancer, namely berbamine, cryptotanshinone, dihydroartemisinin, kadsuphilactone B, methyl lucidone, tanshinone-I, tanshinone-IIA, and tetramethylpyrazine [10–15]. Existing literature indicates that effective plant ingredients may improve the sensitivity of ovarian cancer to multiple drugs when they are particularly used as a preliminary treatment option or as a strategy for sequential therapy, which could further decrease tumor size and metastatic burden of ovarian cancer [16]. *Sophora flavescens* belongs to Sophora genus and the Fabaceae family. It is a well-known traditional Chinese medicine, which has been explored for its potential to mainly treat solid tumors and inflammatory diseases [17]. Also, various bioactive ingredients (like triterpene glycosides, flavonoids, alkaloids, quinones, etc.) of the plants have been used to treat cancers of the breast and lungs, as well as demonstrate anti-inflammatory and antiproliferative properties. These findings have suggested that *S. flavescens* contain bioactive ingredients that have potential antiovarian effect [17]. Kushenol E (KE) is isolated from *S. flavescens* [18, 19] and has been characterized as a biprenylated flavonoid. An extensive search of scientific literature showed that KE has not been explored in the field of discovering effective drugs for the treatment of ovarian cancer. Despite this shortfall, Kwon et al. [18] have reported that KE could inhibit autophagy and impair the positioning of lysosomes in HCT 116 colon cancer and Hela cell lines [18]. In support of the antiovarian potential of KE, Liu et al. [20] discovered that a derivative of KE, Kushenol A (KA), demonstrated an antiproliferative effect in breast cancer. Besides, Yang et al. [21] isolated several prenylated flavonoids with leachianone A, displaying better antiproliferative properties against HepG2 cell lines [21]. Nonetheless, the antitumor applications of the prenylflavonoids in general, including KE, are limited by their poor solubility in water [22], which in turn affects their bioavailability. Till date, nanopreparations to improve the bioavailability of KE are lacking. In view of this gap, ideal strategies to improve the bioavailability and antiovarian potential of KE are urgently needed.

In recent times, scientists have developed various nanosized drug delivery systems, namely nanoparticles [23], micelles, liposomes [24, 25], and nanogels [26] for wide applications, especially tumor targeting [27]. As one of the most successful nanocarriers, biodegradable polymeric micelles have gradually gained attention because they can increase the solubility of hydrophobic drugs in water, improve selectivity, excellent biocompatibility, increase bioavailability, prolonged and sustained circulation time, good in vivo degradability, and accurate accumulation of drugs to tumor site [27, 28]. Herein, the authors selected widely used biodegradable and biocompatibility polymers like block copolymers of poly-(ethylene glycol)-(PEG, PLGA-PEG) and poly-(lactic-co-glycolic acid, PLGA) to prepare nanosized particles for solubility enhancement, and increment of poor bioavailability. Furthermore, cyclic pentapeptide cyclo(-Arg-Gly-Asp-d-Phe-Lys-) (RGDfK) was selected as an active component because of its ability to target tumors. Thus, RGD was specifically conjugated with *α*v*β*3 of *α*v*β*3-integrins, which are highly expressed in ovarian cancer [29] and are key regulators of tumor angiogenesis and progression, including ovarian cancer [30, 31]. During angiogenesis, overexpression of *α*v*β*3-integrins is observed more in tumors of neoendothelial cells and cancerous cells than in most normal cells because they are considered an important hallmark of tumor angiogenesis [32]. Thus, the grafting of nanocarriers with RGD peptide may be regarded as a system of double targeting. In recent times, targeting of nanocarriers with RGD peptide has proven to be favorably important to deliver chemotherapeutics [33, 34], peptides [35], proteins [36, 37], and nucleic acids [38]. Therefore, the conjugation of polymeric micelles with c(RGDfK) peptides to target *α*v*β*3-integrins may be promising therapies for the treatment of ovarian cancer.

This work sought to evaluate the potential of poly(lactic-co-glycolic acid)-poly(ethylene glycol)-modified with cyclic RGDfK peptide (PLGA-PEG-c(RGDfK))-KE micelles (PPCKM) to prenylated flavonoids like KE to ovarian cancer. Thus, this study seeks to prepare and characterize PPCKM using indicators such as particle size, zeta potential (Z-potential), morphology, in vitro release, and critical micellar concentration (CMC). Also, this work aimed to evaluate the antiovarian cancer activity of PPCKM using SKOV-3 cells and xenograft models in BALB/c nude mice.

## 2. Materials and Methods

### 2.1. Chemicals

MedChemExpress (MCE) provided KE (HY-N2463, >96% pure). Toyo Biotech (Shanghai-China) supplied PLGA-PEG-N-hydroxysuccinimide (NHS; PLGA with MW of 4000–6000, 50/50; PEG with MW of 2000) and PLGA-PEG (PLGA with MW of 4000; PEG with MW of 2000), while Beijing Jiangsu Stem Cell Bank (Taizhou, Jiangsu-China) provided SKOV-3 cell line. Also, Gibco BRL (Invitrogen Co., Carlsbad, CA, USA) provided fetal-bovine serum (FBS) and trypsin, RPMI-1640 medium. Fluorescein isothio-cyanate (FITC)-annexin V apoptotic detection kit was outsourced from BD Biosciences (San Jose, CA, USA), which was utilized to investigate the apoptotic activity. Pretreatment of reagent water was performed with a Milli-*Q* plus system (EMD Milli-pore, MA, USA). Chromatographic grade solvents, namely methanol, ethanol, dimethyl sulfoxide (DMSO), and acetonitrile, were used without being treated further.

### 2.2. Methods

#### 2.2.1. Culturing of Cells

The Chinese Academy of Sciences' Cell Bank (Beijing-China) provided cell line of human ovarian adenocarcinoma (SKOV-3). The cells were cultured under atmospheric CO_2_ (5%) in a medium of RPMI-1640 comprising streptomycin (50 μg/mL), penicillin (10 U/mL), and FBS (10%) at 37°C.

#### 2.2.2. Preparation of PPCKM

**Figure 1:**
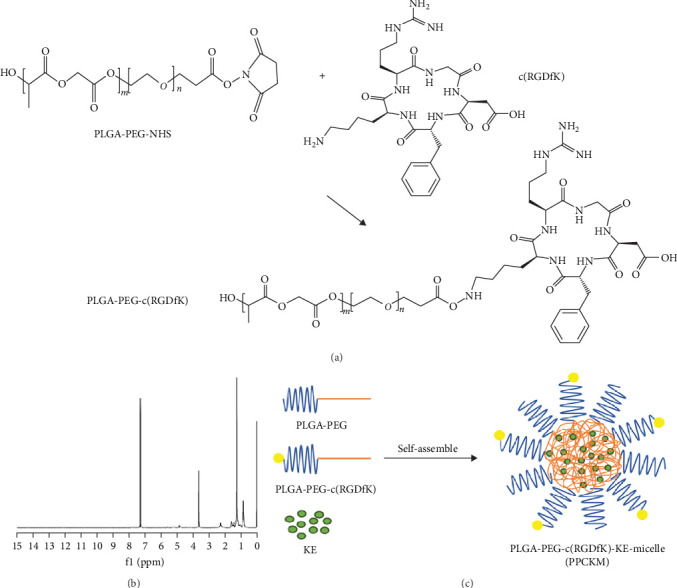
The preparation of PLGA-PEG-c(RGDfK)-Kushenol E-micelle (PPCKM). (A) The synthesis of PLGA-PEG-c(RGDfK). (B) The H1-NMR of PLGA-PEG-c(RGDfK). (C) The diagram of PPCKM preparation. KE, Kushenol E; NMR, nuclear magnetic resonance. PLGA-PEG-c(RGDfK), poly(lactic-co-glycolic acid)-poly(ethylene glycol)-modified with cyclic RGDfK peptide.

PLGA-PEG-c(RGDfK) was prepared according to a procedure employed by Elena de las Heras [39] (Figure 1A) with some modifications. Briefly, PLGA-PEG-NHS (12 μL) was dissolved in a solution of phosphate-buffered saline (PBS, pH = 7.4, 500 μL at 37°C), and peptide c(RGDfK) (24 μmol/L) in DMSO (500 μL). The mixture was shaken before it was allowed to react for 4 h at ambient temperature (20 ± 3°C). The mixture was dialyzed for 24 h in H_2_O (MWCO 2000) prior to freezing of purified solution in liquid nitrogen (N_2_) gas, and it was lyophilized for 24 h. The PLGA-PEG and KE (the molar ratio was controlled at 20:1) were weighed and completely dissolved with ethanol. The mixture was transferred to a round-bottom flask before the ethanol was pumped out with a rotovap device to produce a thin film of PLGA-PEG and the drug. Micelles were formed after PBS (10 mM, pH 7.4) containing PLGA PEG-c(RGDfK) was used to hydrate (the molar ratio of PLGA-PEG-c(RGDfK to PLGA-PEG was 1:100) the film.

#### 2.2.3. Characterization of PPCKM

##### 2.2.3.1. Size of Micellar Particles and Z-Potential

The size of micellar particles, Z-potential, and polydispersity (PDI) index of PPCKM were analyzed via dynamic light scattering (DLS) using the Brookhaven 90 PALS instrument, USA. The PPCKM was diluted with double-distilled water (DDW, 1 : 100 v/v) before it was placed in the cuvette and analyzed at 20°C and an angle of 90°. All the measurements were recorded in triplicate.

##### 2.2.3.2. Morphological Analysis of Micellar Particles With Transmission Electron Microscopic (TEM) Technique

In terms of morphological analysis, PPCKM was observed with TEM (Tecnai-12, Philips, The Netherlands) using a negative staining method [40]. Morphology of PPCKM was observed after the sample had been placed onto mesh copper grids in a drop-wise manner, and excess droplets have also been removed. The samples were stained with a solution (1%, w/v) of phosphotungstatic acid before 15 min of drying at ambient temperature. The samples were subsequently observed with TEM.

##### 2.2.3.3. High Performance Liquid Chromatography (HPLC) Analysis

The samples were analyzed with the HPLC technique through a Shimadzu Scientific instrument that had an LC-20AT pump and detector (an SPD-20A ultraviolet–visible, Shimadzu, Kyoto, Japan). The chromatographic analysis was performed with a solution (65% acetonitrile and 35% water) of acetonitrile flowing at a rate of 0.8% on a C_18_ reverse-phase (5 μm, 250 × 4.6 mm) column and operating at a temperature of 40°C. The concentration of KE was determined through measurement of peak area at 275 nm wavelength and retention time of 17.13 min.

##### 2.2.3.4. Determination of CMC Value

The CMC value of PPCKM was measured using pyrene fluorescence analysis [41] via fluorescence spectrophotometer (RF-5310, Shimadzu Corp, Tokyo, Japan). In brief, PPCKM was diluted to 0.1, 0.5, 1, 5, 10, 50, 100, 500, 1000, and 10,000 µg/mL. A solution of pyrene acetone (90 μL, 4 × 10^−7^ mol/L) was transferred to a 10 mL brown centrifuge tube before acetone was evaporated. Different concentrations of PPCKM (9 mL) were placed in the same above-mentioned tubes. The detection conditions were as follows: 2.5 nm as the widths of excitation slits, 5 nm as the widths of emission slits, and 335 nm as the wavelength of excitation. Meanwhile, 350 and 500 nm were used as the range of emission wavelength. The absorbance (measured at 373 and 384 nm) was recorded, and I373/I384 was calculated. A correlation diagram was obtained from the Origin software 2021.

##### 2.2.3.5. Determination of Encapsulation Efficiency (EE), Loading Efficiency (LE), and Evaluation of Stability

In short, the EE and LE of PPCKM were estimated through the HPLC method as described in a study by Gao et al. [42], with slight modifications. The structure of PPCKM was disrupted using methanol (chromatographic grade) before KE was dissolved and analyzed with the HPLC system. The physical indicators of PPCKM were calculated with Equations (1) and (2).

(1) $$\begin{array}{c} EE\left(\%\right)=\frac{M_{t}-M_{f}}{M_{t}}\times 100\%, \end{array}$$

(2) $$\begin{array}{c} LE\left(\%\right)=\frac{M_{t}-M_{f}}{M_{c}}\times 100\%, \end{array}$$

wherein *M*_*t*_ = total quantity of KE in PPCKM, *M*_*f*_ = quantity of free KE, and *M*_*c*_ = total ingredients in PPCKM.

In addition, storage stability analyses of PPCKM were conducted for 30 days according to a method reported by previous study [42], amid slight modifications. The stability of PPCKM was evaluated by assessing the changes in particle size after the micelles have been stored for 30 days at 25°C.

##### 2.2.3.6. Testing of In Vitro Release of KE From PPCKM

In vitro releases of KE and PPCKM were studied under sink conditions using the dialysis method as stated previously [42, 43]. The release medium for this experiment was mainly a solution of PBS (pH 7.4 mixed with Tween 80 (0.8%, w/v) [44–46]. Briefly, PPCKM solution (containing 1 mg KE) and KE suspension (which was formed by dispersing KE [1 mg] in solution [0.5%] of sodium carboxy-methyl cellulose] were accurately transferred to dialysis bags (with Mw of 3.5 kDa, Genia outsourced from Biotech. Co., Ltd, Beijing-China). The two ends of the dialysis bags were tied before the bags were placed in Erlenmeyer flasks. A controlled shaker (Taicang Hualida Exp. Equip. Co., Ltd., Suzhou-China) was utilized to test the in vitro release at conditions of temperature (37°C) and speed (100 rpm). The dosage forms (1 mL each) were sampled from the releasing medium at predetermined time frames (0.083, 0.17, 0.25, 0.5, 0.75, 1, 1.5, 2, 6, 8, 10, 12, and 24 h), while equal type and volume of releasing medium was used to replenish. The samples were filtered through a hydrophilic membrane filter (0.45 μm) after the performance of centrifugation for 10 min at 1000 rpm. The filtered solution (20 µL) was analyzed with HPLC.

#### 2.2.4. Assaying of Cell Viability

The SKOV3 cells viability was ascertained with 3-(4, 5-dimethylthiazol-2-yl)-2,5-diphenyltetrazolium bromide (MTT) colorimetric assay [47]. The SKOV-3 cells were cultured in 96 well plates at 1 × 10^4^ cells/well density. When the cells reached 60% confluence, KE and PPCKM were added at final concentrations that ranged from 0 to 200 μM (0, 3, 6, 12, 25, 50, 75, 100, 150, and 200 μM) and kept for 12, 24, and 48 h to study the dose- and time-dependent cytotoxic effects. The medium was replaced with MTT after incubation for 12, 24, and 48 h, before further incubation of the cells for 4 h. The MTT-formazan was dissolved in DMSO before the absorbance was measured at 570 nm using an Epoch Microplate Reader (Biotek). The following equation was applied to calculate the cell viability: Cell viability (%) = OD_sample_ /OD_control_ × 100%, where OD_control_ was obtained in the absence of KE and PPCKM, and OD_sample_ was obtained in the presence of KE and PPCKM. The results were expressed as mean ± SD for three replicates.

#### 2.2.5. Apoptotic Rate and Flow Cytometric Analysis

The SKOV-3 cells were treated with various concentrations (0, 15, 30, and 50 μM) of PPCKM and were carried out for 24 h. The cells were then trypsinizated and resuspended in PBS comprising propidium iodide (PI, 0.25 μg/mL). The viable cells (PI-negative) were quantified using the flow cytometric technique [48]. The treated cells were stained with annexin V-FITC for 15 min in annexin V staining buffer before apoptosis was evaluated at ambient temperature and counterstained with PI. The above-mentioned technique was employed to ascertain the apoptotic rate. FlowJo software was used to analyze.

#### 2.2.6. Evaluation of In Vivo Antitumor Efficacy of PPCKM

##### 2.2.6.1. Labeling of PPCKM With CY3 (Cy3-PPCKM)

Based on the existing method [49], PPCKM (1 mg/mL) was labeled with Cy3 to visualize its accumulation in tumor using mouse model of ovarian tumor. After conjugation of PLGA-PEG-NHS to peptide c(RGDfk), the resultant compound was further reacted with Cy3 dye. Peptide conjugate (1 mL) was diluted with PBS (10 mM, pH 7.4) to obtain a final concentration (1000 µg/mL). The Cy3 dye stock solution (10 μL, 1 mM) was incubated for 30 min with peptide conjugate at ambient temperature. The unreacted Cy3 was removed from the solution through dialysis after incubation.

##### 2.2.6.2. Experimental Animals and Xenografts Model

Research Center for Laboratory Animal at Jiangsu University supplied BALB/c (nu/nu) nude mice (female, 180–240 g). The mice were maintained (for 1 week) under controlled conditions, namely 25 ± 2°C (RH−55 ± 5%) and a cycle of 12 h-light: 12 h-dark with unrestricted access to water and food. The committee for the care and use of laboratory animals at Jiangsu University gave approval for this study. With RPMI-1640 as a medium, a single SKOV-3 cells suspension (1 × 10^7^ cells/mL) was prepared before injection (200 μL) of the cells into the right side of each mouse's back via the subcutaneous route. The tumors developed after 1 month (average volume of tumor was 300 ± 18.5 mm [3]), which successfully established 32 SKOV-3 xenograft models [50]. The mice (*n* = 32) were allotted randomly to four groups (*n* = 5), viz., (1) Model (normal saline with Cy-3 content equal to PPCKM group), (2) KE (10 mg/kg, with Cy-3 content equal to PPCKM group), (3) PPCKM (10 mg/kg, with Cy-3 content equal to PPCKM group), and (4) PPCKM groups (10 mg/kg, labeled by Cy-3). During the period of treatment (2 weeks), the drugs were administered intraperitoneally every 3 days.

##### 2.2.6.3. Growth of Tumor

During the treatment period, the average volume (*V*) of the tumor was calculated daily with the appropriate equation:

*V* = *a*^⁣^*∗*^^*b*^⁣^*∗*^^*b*/2 (where *a* denotes maximal tumor diameter, *b* represents the maximal vertical diameter of tumor). The curves of tumor growth were constructed based on the tumor volume. Antitumor effectiveness of the treatments was measured in terms of tumor-growth inhibition (TGI) through a method described in previous work [51]. The TGI was calculated with Equation (3):

(3) $$\begin{array}{c} TGI=\left[\left(1-\frac{T}{C}\right)\times 100\right]\%, \end{array}$$

where *C* denotes the final mean tumor weight of mice in the model group, and *T* indicates the final mean tumor weight of mice in the treatment group.

##### 2.2.6.4. Optical Imaging

With regard to ovarian tumor mouse models, the experiment was designed in order to envision the potential of PPCKM to accumulate tumors. Thus, performed optical imaging was performed using an in vivo Xtreme II imaging system (Bruker, Germany). The optical imaging was carried out under parameters with 500–550 nm and 570–600 nm as the respective excitation and emission filter wavelengths. A vendor software was applied to determine the quantitative nanoparticles distribution in main organs via measurements of fluorescence. The average of each organ's fluorescence intensity per square inch was measured before it was subtracted from the value of control.

##### 2.2.6.5. Immunohistochemical Analysis

The PPCKM effect on in vivo proliferating cell nuclear antigen (PCNA) and Ki67 expressions in tumor grafts was assessed with immunohistochemical analysis. The tumor grafts were fixed in para-formaldehyde (4%), before they were embedded in paraffin, and sliced into sections (4 μm). In the nucleus, the authors observed that PCNA and Ki67-positive cells were stained brown. In a blind fashion and under a high-power field (200x magnification), the proportion of PCNA and Ki67-positive cells were counted in areas that have been randomly selected (*n* = 10) in an objective grid. The extent of PCNA and ki67 expressions was quantified via labeling index and computed using ImageJ.

##### 2.2.6.6. Terminal Deoxy-Nucleotidyl-Transferase-Mediated 2'-Deoxyuridine 5'-Triphosphate (dUTP)-Nick End-Labeling (TUNEL) Assay for Apoptotic Detection

Apoptosis in the mouse model of SKOV-3 xenograft was determined through the TUNEL assay. Based on the specifications of the manufacturer, the assay was performed with a one-step TUNEL apoptotic kit (Beyotime-China).

#### 2.2.7. Statistical Analysis

Experimental results were presented as mean and standard deviation. Experimental data were statistically analyzed after they have been tested for homogeneity and normal distribution. Statistical methods like independent samples *t*-tests, Fisher's least-significant difference test, and one-way analysis of variance (ANOVA) were utilized to analyze differences within two or more groups. The entire analyses were performed with SPSS software (version 19.0; SPSS, Chicago, IL-USA), wherein a significant level was acceptable at *p* < 0.05.

## 3. Results

### 3.1. Characterization of PPCKM

The c(RGDfK)-modified targeted micelles were successfully prepared to deliver KE to ovarian cancer sites. The PLGA-PEG-NHS and c(RGDfK) were combined via vibration at 20 ± 3°C (Figure 1A). The chemical structure of PLGA-PEG-c(RGDfK) was confirmed using proton (1 H) nuclear magnetic resonance (NMR) (Figure 1 B). The peaks at 7.0–8.0 ppm belonged to the typical protons of c(RGDfK), while the peaks at 3.66 ppm belonged to the typical protons of PLGA-PEG. The yield of PLGA-PEG-c(RGDfK) was 74.03% ± 1.01%. Micellization (PPCKM) of PLGA-PEG-c(RGDfK), PLGA-PEG, and KE was performed via film dispersion (Figure 1C), which was modified through condensation of Cy3-COOH with PLGA-PEG-NHS-c(RGDfK)-Guanido for tracing in vivo.

To determine the concentration of KE in vitro, the HPLC analytical method was established with the standard curve as *Y* = 74652*X* + 108378, *R*^2^ = 0.999 (Table 1). The results of intraday/interday precision, recovery test, stability, and repeatability are shown in Tables 2–6.

**Figure 2:**
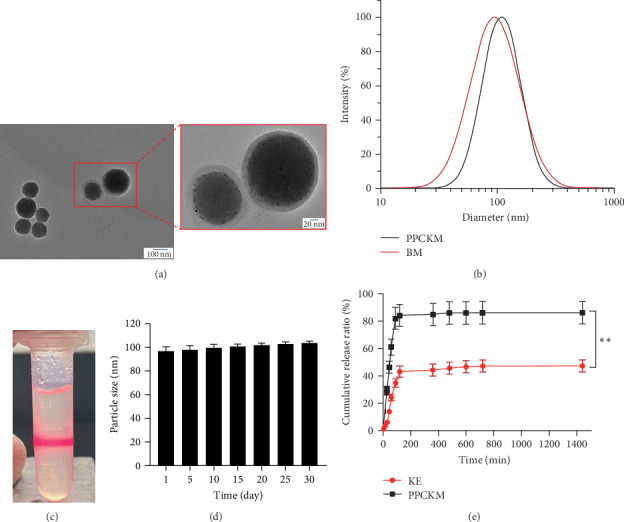
The characteristics of PLGA-PEG-c(RGDfK)-Kushenol E-micelle (PPCKM). (A) The result of transmission electron microscopy. (B) Particle size. (C) Appearance. (D) Storage and stability analysis. (E) The cumulative release. BM, blank micelles; KE, Kushenol E; PLGA-PEG-c(RGDfK), poly(lactic-co-glycolic acid)-poly(ethylene glycol)-modified with cyclic RGDfK peptide. *⁣*^*∗∗*^*p* < 0.01 (vs. KE group).

After the development of the micelles, they were appropriately characterized using indicators such as morphology, hydrodynamic size, PDI, Z-potential, appearance, CMC, storage stability, EE, and LE. The micellar particles of PPCKM were discovered to be spherically shaped (Figure 2A), while the hydrodynamic size and PDI were the same as that of the findings of NanoBrook 90Plus PALS (namely 93.41 ± 2.84 nm, PDI = 0.285 ± 0.040, Table 7) result. The particle size of blank micelles (BM) was 95.43 ± 1.10 nm (PDI = 0.261 ± 0.004), which was not significantly different when compared with PPCKM (Figure 2C). In terms of appearance, the nanomicelles appeared as a homogeneous light-yellow solution (Figure 2B). The Z-potential of PPCKM and BM was −6.68 ± 0.37 mV and −8.12 ± 1.10 mV, respectively (Table 7). Also, the CMC of PPCKM was estimated to be 0.00912 mg/mL (Figure 2D). Following storage of PPCKM (Figure 2E) at 25°C, changes in particle size at 1, 5, 10, 15, 20, 25, and 30 days did not differ significantly different (*p* > 0.05) as indicated by student *t* test. Furthermore, the respective EE and LE of PPCKM in water (obtained via the Milli-*Q* system) were 89.7% ± 1.3% and 5.93% ± 0.5%. After KE had been loaded into micelles, the solubility of PPCKM in PBS (containing 0.8% Tween 80) showed a significant improvement (*p* < 0.01, Figure 2F).

### 3.2. Inhibition of SKOV-3 Cells Viability by PPCKM

This experiment was designed to detect the effect of PPCKM on the survival of SKOV-3 cells. At different durations (24, 48, and 72 h), the SKOV-3 cells were treated with distinct PPCKM concentrations (0, 3.13, 6.25, 12.50, 25.00, 50.00, 100.00, 150.00, and 100.00 μM). The respective IC_50_ of KE and PPCKM were 33.39 and 18.56 μM at 24 h, 36.16 and 20.93 μM at 48 h, and 32.34 and 17.77 μM at 72 h (Figure 2A–C). Therefore, the value of IC_50_ was substantially decreased by PPCKM (*p* < 0.05) compared to that by KE. PPCKM inhibited the viability of SKOV-3 cells dose-dependently. In subsequent experiments, concentrations (15, 30, and 50 μM) of PPCKM were studied at 24 h.

### 3.3. PPCKM Restrained Invasion and Migration of SKOV-3 Cells

**Figure 3:**
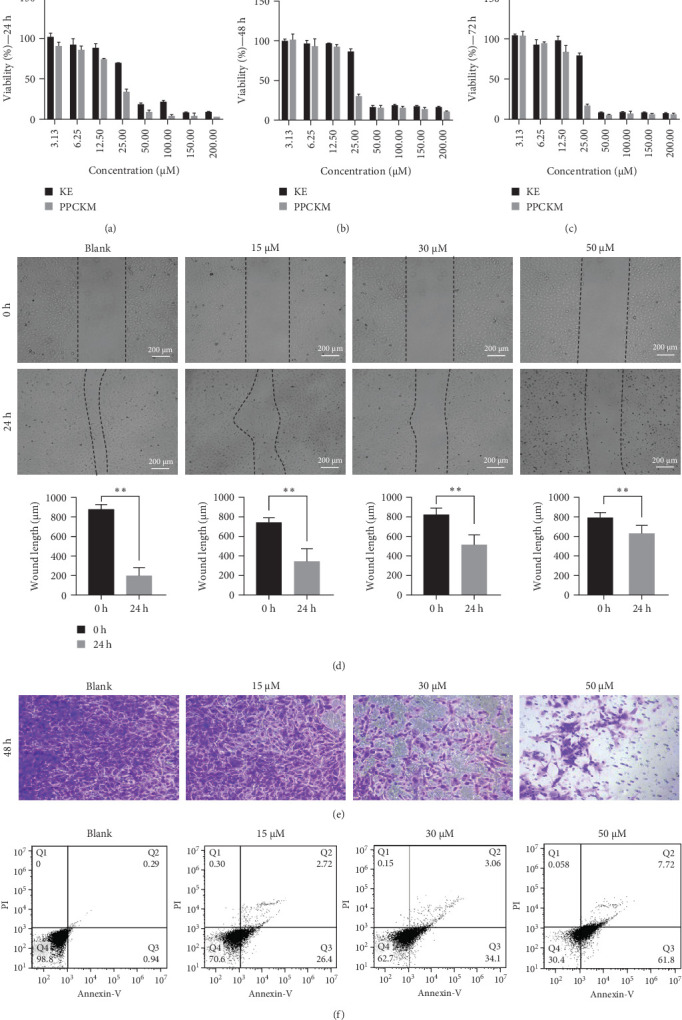
The antitumor effect of PLGA-PEG-c(RGDfK)-Kushenol E-micelle (PPCKM) in vitro. (A–C) The viability of SKOV-3 cells under KE and PPCKM different concentrations. (D) The migration results. (E) The invasion results. (F) Apoptosis rate and flow cytometry analysis. KE, Kushenol E; PI, propidium iodide; PLGA-PEG-c(RGDfK), poly(lactic-co-glycolic acid)-poly(ethylene glycol)-modified with cyclic RGDfK peptide. *⁣*^*∗∗*^*p* < 0.01 (vs. 24h).

The effect of PPCKM on the invasion and migration of SKOV-3 cells was investigated using the Transwell and scratch assays. One-way ANOVA demonstrated a marked decrease in wound length (1.3–4.5 times) compared with that of 0 h (*p* < 0.01, Figure 3D). However, the rate of decline in wound length was slowed after treatment with PPCKM (50 μM). Consistent results were discovered between scratch and Transwell assays (Figure 3E).

### 3.4. PPCKM Induced Apoptosis in SKOV-3 Cells

Apoptotic analysis showed that PPCKM promoted in vitro early and late-phase (Annexin V^+^/PI^−^) apoptosis in SKOV-3 cells (Figure 3F) compared to the blank group. A marked increased percentage of apoptotic cells was observed in 50 μM batch compared with 15 and 30 μM groups.

### 3.5. PPCKM Decreased Tumor Volume, Weight, and TGI in a Mouse Model of SKOV-3 Xenograft

**Figure 4:**
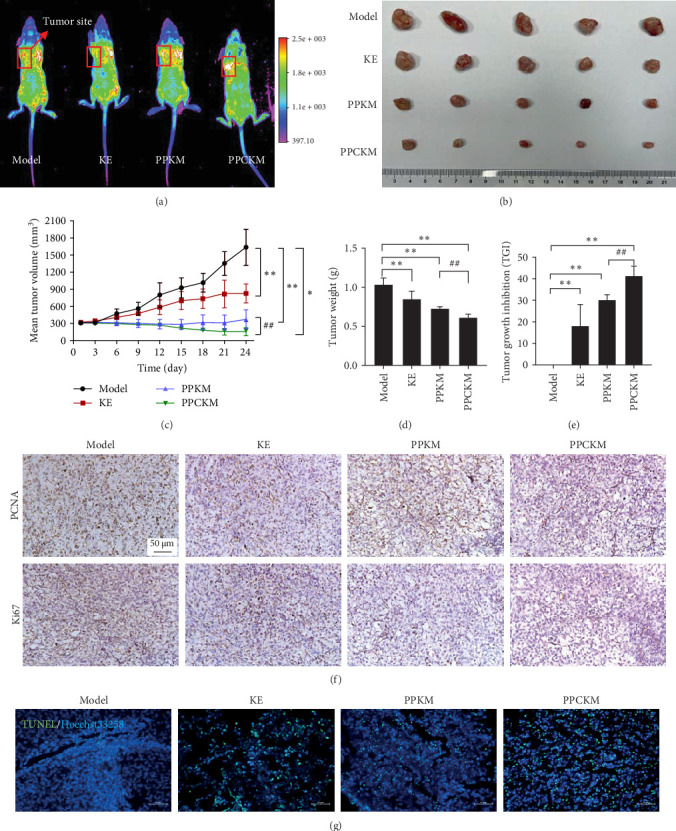
The antitumor effect of PLGA-PEG-c(RGDfK)-Kushenol E-micelle (PPCKM) in vivo. (A) The image of PPCKM under Bruker in vivo Xtreme II imaging system. (B) The tumor size of model, KE, PLGA-PEG-KE micelles (PPKM), and PPCKM groups. (C) The tumor volume change. (D) The change of tumor weight. (E) The change of tumor growth inhibition. (F) The expression of PCNA and Ki67 by IHC. (G) The TUNEL staining. IHC, immunohistochemistry; KE, Kushenol E; PCNA, proliferating cell nuclear antigen; PLGA-PEG-c(RGDfK), poly(lactic-co-glycolic acid)-poly(ethylene glycol)-modified with cyclic RGDfK peptide. *⁣*^*∗*^*p* < 0.05, *⁣*^*∗∗*^*p* < 0.01 (vs. model group), ## *p* < 0.01 (PPCKM vs. PPKM).

The intensity of Cy-3 in the model, KE, and PLGA-PEG-KE micelles (PPKM) groups mainly accumulated in the lungs and kidneys, but little fluorescence was detected in tumors marked with red (Figure 4A). However, the Cy-3 intensity was found to be the highest at the tumor site after treatment with PPCKM. The tumor size in the PPCKM group was markedly reduced (Figure 4B). The growth curves (Figure 4C) depended on the average volume of tumor. The tumors of mice in the model group were discovered to grow quickly. However, in the KE group, the tumors of the mice grew slowly in size and volume, which displayed a lower average tumor volume (Figure 4C). During the first 12 days, tumor volume of mice in the PPKM group remained almost unchanged but slowly increased thereafter. Likewise, the tumor volume of mice in the PPCKM group remained unchanged on the same days as stated above, but thereafter, it decreased slowly. Also, the volume of tumor was decreased by PPKM and PPCKM, amid the demonstration of similar growth curves during the first 12 days. One-way ANOVA displayed that the PPCKM group exhibited the lowest average tumor volume (*p* < 0.01). The tumor weight decreased in the three treated groups compared to that in the model group (Figure 4D). One-way ANOVA demonstrated that the tumor weights were lowered substantially in mice in the PPCKM group compared to KE and PPKM groups (*p* < 0.01). The KE, PPKM, and PPCKM produced TGIs of 17.99%, 29.98%, and 41.20%, respectively (Figure 4E).

### 3.6. PPCKM Downregulated PCNA and Ki67 and Promoted Apoptosis In Vivo

In the in vivo studies, PCNA and Ki67 expressions were affected by KE, PPKM, and PPCKM (Figure 4F). The PPCKM inhibited PCNA and Ki67 expressions in the ovarian cancer mice. In contrast, KE did not have any effect on PCNA and Ki67 expressions. TUNEL staining was observed in vivo (Figure 4G). The KE, PPKM, and PPCKM groups demonstrated substantial upregulation of TUNEL (FITC+) expression compared to the model group.

## 4. Discussion

The mortality rate of ovarian cancer is high because the disease is diagnosed in the advanced stage because of factors such as delayed onset of symptoms, asymptomatic and secret growth of tumors, and lack of appropriate screening tests [4]. The recurrence rate of ovarian cancer is as high as 80% at 1–2 years after chemotherapy, with considerable side effects. Available literature has suggested that the high recurrence rate of ovarian cancer is owing to the inherent chemoresistance of the disease, which may be due to drug-resistant cells and reduced immunosurveillance [52]. Also, the inherent chemoresistance of ovarian cancer is probably due to cancer stem cells, which are considered biologically distinct remaining drug-resistant cells [53]. Therefore, leveraging on high efficacy and low toxicity targeted delivery drug systems may improve the treatment of ovarian cancer.

During angiogenesis, overexpression of *α*v*β*3-integrins is observed more in tumors of neoendothelial cells and cancerous cells than in most normal cells because they are considered an important hallmark of tumor angiogenesis. The sequence of RGD is potentially conjugated with *α*v*β*3-integrins, with the former binding the latter with a high affinity [54]. To ensure active targeting of tumors, the nanocarriers are modified with RGD such that they particularly target drugs to tumor cells and/or angiogenic endothelial cells via RGD peptide binding to *α*v*β*3, which is overexpressed by the above-mentioned cells [55]. As positive tumorigenetic regulators, *α*v*β*3-integrins targeting nanoparticles have been developed in recent times by scientists to target tumor cells with RGD peptide as ligands [56, 57]. Lv et al. [58] fabricated nanoparticles with RGD peptides as ligands that potentially targeted tumor cell lines (like MCF-7, C26, and C6 cells) that overexpressed the *α*v*β*3-integrins. Egorova et al. [59]. used the same concept to develop nanoparticles of peptide with *α*v*β*3-integrins as the targeting agent, which specifically delivered deoxyribonucleic acid to uterine tumor cells. Scientists have revealed through fluorescence probes that cells of ovarian cancer and surfaces of the tumor blood vessels highly express *α*v*β*3-integrins, which enabled targeting of the tumor (i.e., OVCAR-4 cell line) [60]. Thus, ovarian cancer can be targeted with micelles by coupling compounds with anticancer potential to the RGD peptides [61, 62].

Prenylated flavonoids of *S. flavescens* have been shown to possess various pharmacological properties such as antioxidative, anti-inflammatory, and anticancer effects [21, 63]. Derivatives of KE, Kushenol Z (KZ), and KA have been proven to respectively induce apoptosis in cancer cells of non-small cell lung [64] and breast [20]. More importantly, KE has been found to inhibit autophagy [18], which has been exploited for the treatment of ovarian cancer [65, 66]. Nonetheless, the anticancer effect of KE has not been maximized because of its poor solubility in water. This drawback affects the circulation time and bioavailability of the hydrophobic flavonoid, possibly through its inability to penetrate the plasma membranes [67]. In this scenario, nanosized delivery systems are the ideal strategy to increase the solubility of the flavonoid by reducing the particle size and increasing the specific surface area for the enhanced rate of dissolution and solubility [68]. Smaller-sized nanoparticles are able to reach the site of tumor cells in close proximity via blood circulation by passing through the wall of capillary and stroma of dense tumor to interact with tumor cells [69]. Herein, this study leverages on the advantages of the above-mentioned concept to prepare PPCKM.

The prepared PPCKM and BM had particle sizes of 93.41 ± 2.84 nm and 95.43 ± 1.10 nm. The hydrodynamic particle sizes of the blank and KE-loaded micelles were statistically similar, which agrees with existing literature [70] and suggests that the incorporation of KE may not have disrupted micellar structure. Available report has indicated that micelles with particle size below 100 nm could decrease nonselective targeting of drugs to normal cells but promote substantial endocytosis and permeability by ovarian cancer cells [71]. In terms of morphology, PPCKM displayed a spherical shape, which suggests that the micelle may be readily taken up by ovarian cancer cells [72]. The study has revealed that PPCKM displayed Z-potential was −6.68 ± 0.37 mV, which fell within a similar range that was observed in previous work [73] and did not vary significantly compared with BM [74]. Although the Z-potential was not sufficiently high, its stability was satisfactory. Thus, storage stability evaluation of the micelles showed that changes in particle size at 1, 5, 10, 15, 20, 25, and 30 days did not statistically differ. The hydrophobic-hydrophilic nature of the PLGA-PEG copolymer may account for the observed above findings. The copolymer has been found to produce smaller-sized nanoparticles with increased stability and sustained time of drug circulation [75]. In support of the above observations, the study revealed that the CMC of PPCKM was far lower than the low-molecular-weight surfactant in water. This finding suggests that PPCKM was stable and highly resistant to micellar dissociation upon dilution. The solubility of PPCKM in PBS showed an obvious improvement, which may be ascribable to the hydrophilic portion of the micelles. Importantly, PEGylation has been shown to enhance the aqueous solubility of lipophilic drugs via an increase in the molecular weight of drug molecules [76]. The PPCKM preferentially targeted tumor cells through fenestrations [32] by directly accessing the vasculature of the tumor cells owing to its small particle size. Usually, modification of nanoparticles with appropriate ligands ensures precise interaction between these particles and tumor cells through their plasma membranes and micro-environment, thereby leading to delivery of increased drug content to tumor site [77]. The results suggest that the PPCKM had obvious anti-ovarian cancer effect. To further understand the effect of PPCKM on ovarian cancer, the team measured the viability, invasion capacity, migration capability, and apoptotic rate of SKOV-3 cells under PPCKM treatment. The results showed that PPCKM significantly decreased the IC_50_, inhibited invasion and migration, as well as enhanced SKOV-3 cells apoptosis compared to KE. This differential anti-ovarian cancer effect may be ascribable to the presence of RGD on the surfaces of PPCKM. In agreement to this finding, Jin et al. [78] discovered that RGD-ligand pH-sensitive micelles loaded with lonidamine or betulinic acid and doxorubicin could potentially target ovarian cancer. Likewise, RGD-based albendazole-loaded micelles that were prepared by Zhao et al. could be readily taken up by ovarian cancer cells [79]. Again, in support to our findings, Long et al. [80] developed nanoparticles modified with RGD to precisely target and suppress ovarian tumors growth. The antitumor effect of PPCKM may have been increased as a result of the potential of RGD to particularly recognize integrin receptors of ovarian tumor cells membrane [81].

In vivo study using a xenograft model in BALB/c nude mice confirmed the potential of PPCKM to target ovarian cancer. Conjugation of PPCKM with Cy-3 and imaging affirmed that more PPCKM was delivered to the tumor site after comparison with the intensity of Cy-3. This observation demonstrates the tumor-targeting ability of PPCKM. These findings confirm those of earlier studies, which designed nanoparticles for drug delivery via grafting with RGD peptide [82–84]. In this work, RGD-loaded micelles exhibited therapeutic efficacy by effectively retarding the growth of the tumor and prolonging the survival times of the xenograft mice compared to KE. This finding was further supported by the obvious decrease in tumor volume, weight, and TGI in the PPCKM-treated group compared to the other groups. Also, after 2 weeks of treatment, biodistribution analysis showed that the Cy-3-labeled PPCKM accumulated mainly in the tumor. Moreover, a small amount of fluorescence was detected in the right kidney and liver, which further indicates that RGD potentially enhanced the targeting ability of the micelle to tumor. In an attempt to study, the mechanism of PPCKM, the expression of PCNA and Ki67 that was detected with TUNEL assay showed that PPCKM inhibited PCNA and Ki67 expression but upregulated the TUNEL (FITC+) expression compared to the model group. PCNA and Ki67 are the markers of ovarian cancer, with the former predicting the proliferation of malignant epithelial tumor of the ovary [85]. These findings suggest that PPCKM may treat the proliferation of ovarian cancer through the inhibition of PCNA and Ki67 expressions. This study has obviously provided worthful insight into how PPCKM can be potentially applied as a treatment option for ovarian cancer, but it is subject to various limitations. Presently, scientists cannot understand the precise mechanism of the micelle because of the limited scope of markers that were ascertained in this work. Hence, investigation of signaling pathways in ovarian cancer that can be modulated by PPCKM would provide valuable information for future applications of the micelle. Also, the evaluation of the effect of PPCKM on the pharmacokinetics of KE was not studied. To understand how PPCKM circulates in the bloodstream, future study should address this limitation. Moreover, the effect of PPCKM on noncancerous ovarian cells and adjacent normal cells was not evaluated. Future applications of the micelle should prioritize investigation of the toxicity of PPCKM, which is an important aspect of drug discovery. Because there is a variation of integrin expression within different types of tumors and even among the same types of individual cancers, further animal tumor models are needed to comprehensively establish and demonstrate the targeting ability of PPCKM. PPCKM should be further studied to address these limitations before future applications in the clinics.

## 5. Conclusion

Herein, PPCKM were fabricated with PLGA-PEG copolymer as the hydrophobic-hydrophilic anchor and RGD as integrin receptor ligands. Physical characterization of PPCKM showed acceptable characteristics regarding the size of nanoparticles, Z-potential, and EE. The PPCKM nanoplatform could improve the solubility of KE and enhance its ovarian tumor-targeting effect. The results presented in this study demonstrate the anticancer potential of KE and exhibit the tumor-targeting effect of PPCKM, particularly in ovarian cancer. The finding indicated herein provides a valuable foundation for further development of PPCKM as a novel approach to target ovarian cancer and possibly other tumors.

## Figures and Tables

**Table 1:** Linear test between KE concentration and peak area in vitro.

Concentration (μg/mL)	2.5	5	7.5	15	25	32.5	50	100	200

Peak area	229,456	509,192	671,000	1,291,218	1,698,700	2,737,506	3,848,660	7,658,788	14,990,972

Abbreviation: KE, Kushenol E.

**Table 2:** Intraday precision results of various concentrations of KE (*n* = 5)

Sample	Measured concentration (μg/mL)					Mean	RSD
(μg/mL)	1	2	3	4	5	(μg/mL)	(%)
5	5.02	5.04	4.96	5.02	4.98	5.00	0.66
25	24.99	25.05	25.05	24.86	24.94	24.98	0.32
125	125.52	124.61	124.58	126.43	124.38	125.10	0.69

Abbreviations: KE, Kushenol E; RSD, relative standard deviation.

**Table 3:** Interday precision results of various concentrations of KE (*n* = 5)

Sample	Measured concentration (μg/mL)					Mean	RSD
(μg/mL)	1	2	3	4	5	(μg/mL)	(%)
5	4.97	4.84	5	5.1	5.03	4.99	1.92
25	25.04	24.51	24.66	25.33	25.25	24.96	1.44
125	123.92	127.02	123.49	125.71	126.47	125.32	1.24

Abbreviations: KE, Kushenol E; RSD, relative standard deviation.

**Table 4:** Recovery test of various concentrations of KE (*n* = 3).

Sample	Recovery (%)			Mean	RSD
(μg/mL)	1	2	3		(%)
5	99.19	100.83	99.9	99.97	0.82
25	100.59	99.8	98.84	99.74	0.88
125	99.55	101.1	99.99	100.21	0.80

Abbreviations: KE, Kushenol E; RSD, relative standard deviation.

**Table 5:** Results of stability of various concentrations of KE (*n* = 3)

Time (h)	Average measured value (μg/mL)		
	5	25	125
0	5.08	25.19	126.05
2	5.07	25.12	125.64
4	5.06	25.08	125.29
8	5.04	25.06	125.07
12	4.98	24.97	124.94
24	4.94	24.77	124.77
48	4.92	24.69	124.54
RSD (%)	1.31	0.75	0.42

Abbreviations: KE, Kushenol E; RSD, relative standard deviation.

**Table 6:** Repeatability test of various concentrations of KE (*n* = 5).

Sample	Measured concentration (μg/mL)					Mean	RSD
(μg/mL)	1	2	3	4	5	(μg/mL)	(%)
5	5.04	4.98	4.97	4.99	5	5.00	0.54
25	25.18	24.8	24.86	25.01	25.24	25.02	0.77
125	125	125.22	126.24	124.71	124.61	125.16	0.52

Abbreviations: KE, Kushenol E; RSD, relative standard deviation.

**Table 7:** Particle size and zeta potential of BM and PPCKM (*n* = 3).

Formulation	Particle size (nm)	PDI	Zeta potential (mV)
BM	95.43 ± 1.10	0.261 ± 0.004	−8.12 ± 1.10
PPCKM	93.41 ± 2.84	0.285 ± 0.040	−6.68 ± 0.37

Abbreviations: BM, blank micelles; PDI, polydispersity; PPCKM, poly(lactic-co-glycolic acid)-poly(ethylene glycol)-modified with cyclic RGDfK peptide (PLGA-PEG-c(RGDfK))-KE micelles.

## Data Availability

The collected data files are available on request from the corresponding author.
